# Research gaps in neonatal HIV-related care

**DOI:** 10.4102/sajhivmed.v16i1.375

**Published:** 2015-05-20

**Authors:** Mary-Ann Davies

**Affiliations:** 1 Centre for Infectious Disease Epidemiology and Research, School of Public Health and Family Medicine, University of Cape Town, South Africa

## Abstract

The South African prevention of mother to child transmission programme has made excellent progress in reducing vertical HIV transmission, and paediatric antiretroviral therapy programmes have demonstrated good outcomes with increasing treatment initiation in younger children and infants. However, both in South Africa and across sub-Saharan African, lack of boosted peri-partum prophylaxis for high-risk vertical transmission, loss to follow-up, and failure to initiate HIV-infected infants on antiretroviral therapy (ART) before disease progression are key remaining gaps in neonatal HIV-related care. In this issue of the *Southern African Journal of HIV Medicine*, experts provide valuable recommendations for addressing these gaps. The present article highlights a number of areas where evidence is lacking to inform guidelines and programme development for optimal neonatal HIV-related care.

## Research gaps in neonatal HIV-related care

The South African (SA) prevention of mother to child transmission (PMTCT) programme has made excellent progress in reducing vertical HIV transmission.^1,2^ In addition,^1^ paediatric antiretroviral therapy (ART) programmes have demonstrated good outcomes with increasing treatment initiation in younger children and infants, in response to South African and World Health Organization (WHO) guidelines expanding recommendations for immediate ART from infants to all children < 5 years old.^2,3,4,5,6,7,8,9,10^ However, both in SA and across sub-Saharan African, lack of boosted peri-partum prophylaxis for high-risk vertical transmission, loss to follow-up (LTFU) for early infant diagnostic (EID) HIV-polymerase chain reaction (PCR) testing and receipt of results, and failure to initiate HIV-infected infants on ART before disease progression are key remaining gaps in neonatal HIV-related care.^11,12,13^ The expert reviews on recognising and managing vertical transmission risk in the peri-partum period,^14^ HIV diagnostic testing of newborns^15^ and provision of neonatal ART^16^ provide valuable recommendations for addressing these coverage gaps. They also highlight a number of areas where evidence is lacking to inform guidelines and programme development for optimal neonatal HIV-related care.

### What is the best way to manage newborns at increased risk of intrapartum HIV transmission?

For many years, guidelines from developed countries^17,18,19^ included identification of neonates at high risk of vertical transmission,^20,21^ recommending multi-antiretroviral (multi-ARV) post-exposure prophylaxis (PEP) for these neonates. While two studies provide direct evidence that multi-ARV PEP is more effective than a single drug,^22,23^ data are lacking on the best choice, number and duration of drugs.

Research is needed to inform guidelines that balance the prophylactic benefit of multi-ARV PEP with risks of toxicity and resistance that may increase with duration, as well as the programmatic challenges associated with effectively implementing more complex regimens for longer periods. Furthermore, longer-duration multi-ARV PEP may suppress HIV viral load,^23,24,25^ making HIV diagnosis more challenging, with implications for EID algorithms. In wealthy countries with low maternal HIV prevalence, reduced sensitivity of HIV-PCR is of less concern as guidelines recommend EID testing at numerous time points in exposed infants. In contrast, in South Africa, routine testing in otherwise well infants will probably be restricted to two HIV-PCR tests per infant at most, hence the impact of reduced sensitivity of HIV-PCR testing is critical.

Research needs include determining the effectiveness of routine programmes in accurately identifying high-risk infants in need of multi-ARV PEP, as well as retention, adherence and transmission in routine care. For example, monitoring and evaluation of the implementation of the National and Western Cape provincial guidelines for multi-ARV PEP could provide valuable data to inform programme development.

### What are the optimal algorithms for early infant diagnostic?

South African 2014 PMTCT guidelines provide for HIV-PCR testing at birth of HIV-exposed infants at high risk of vertical HIV transmission, including low-birthweight and premature infants as well as those born to mothers on ART for < 4 weeks or with HIV-RNA > 1000 copies/mL.^26^ In addition, a WHO technical expert panel in 2013 reviewed the optimal timing of EID testing, and consideration was given to recommending either universal or risk-based birth testing.^27^ Given the shift towards a greater proportion of in utero infections with improved coverage of more effective PMTCT regimens such as Option B/B+, and the proven efficacy of HIV-PCR at birth to identify 75% of infections detectable by 6 weeks of age, birth EID could be pivotal in mitigating against the high LTFU for EID testing and delays in starting infected infants on ART.^23,28^ However, there are a number of questions regarding optimal EID guidelines.

#### What is the best time for a follow-up HIV-polymerase chain reaction test?

Birth testing necessitates a follow-up test, as 25% of early HIV infections are undetectable at birth.^23,28^ Evidence is needed to inform the optimal timing of follow-up testing, which depends on test performance, morbidity and mortality without ART (and the effect of ART in reducing this), retention strategies and operational considerations of aligning follow-up with routine child health visits.^27,29^ Whilst mathematical modelling suggests that, with two HIV-PCR tests per infant, the greatest number of infections can be identified at birth and 10 weeks of age, there is a need for more evidence to inform the model assumptions, such as the probable loss of HIV-PCR sensitivity owing to nevirapine PEP.^30^ Of note, no studies to date have examined HIV-PCR test performance at birth and thereafter, now that triple ART is mandated for all women during pregnancy and breastfeeding together with extended infant NVP prophylaxis. These factors may all reduce test sensitivity at birth^31^ and at 6 weeks of age.^25^ For this reason, the 2014 South African PMTCT guidelines defer PCR testing to 16 weeks of age in infants who need 12 weeks of NVP PEP.^26^ However, more data are needed on the duration and extent of reduced sensitivity post-PEP.^24^ Delaying testing will improve sensitivity; however, attrition is likely to increase at longer post-partum durations.^32^ The extent to which such attrition would be exacerbated by false reassurance of a negative result at birth is unknown.

In addition, the model assumes that ART very early in life is associated with improved survival. However, there are little data on the magnitude of the mortality benefit when ART is initiated very early, for example in the first days or weeks of life, especially in premature low-birthweight infants, compared with the benefit seen in the Children with HIV Early antiRetroviral (CHER) trial where the median age of ART initiation in the early group was 7.4 weeks.^33^

#### Is it efficient to test all infants routinely at birth, or should this be restricted to those at high risk of transmission?

Whilst the mathematical modelling study demonstrates a nearly 50% increase in the number of life years saved when adding a routine birth test to a single test at 10 weeks of age, more tests increase costs and reduce efficiency (measured in terms of new diagnoses per PCR) by approximately 35%.^30^ The efficiency of restricting birth testing to those at high risk of transmission is unknown and may be programmatically more difficult to implement effectively. In particular, more data would be needed to identify easily implementable high-risk criteria that are predictive of a high likelihood of a positive birth test.

Although the specificity, and therefore positive predictive value (PPV) of currently used EID tests is very high, the proportion of false-positive tests increases with reduced transmission rates. Follow-up confirmatory testing with HIV-PCR or viral load may be difficult to interpret in infants on prophylactic therapy,^25^ with careful counselling needed. Research is needed on how best to manage patients with false-positive or indeterminate test results, which may be resource-intensive and difficult outside specialist facilities.

#### How feasible is implementation of birth testing in routine care settings?

EID testing at 6 weeks and follow-up for results at the 10-week immunisation visit is currently performed at immunisation clinics. For a birth PCR, delivery facilities would be responsible for birth testing. The very high proportion of facility-based deliveries in SA favours birth testing, and routine postnatal follow-up within a few days of birth at many delivery facilities could facilitate receipt of results. However, the extent to which the additional workload can be absorbed by delivery facilities is unclear. This consideration is particularly important for high-burden facilities where women may be discharged within hours of delivery so that counselling and birth tests may need to be performed after hours or on weekends. There are encouraging early data from a pilot study comparing birth testing of high-risk infants in primary (Khayelitsha, Cape Town) and tertiary (Tygerberg Hospital, Cape Town) care delivery facilities, supporting the feasibility of implementing birth testing at primary care level.^34^ There was no difference in median time-to-test results between the facilities despite no on-site laboratory at the primary care facility.^34^ However, additional research staff supported the study and testing was limited to high-risk infants (about 25% of all exposed infants), therefore the results may not be generalisable to all infants in routine care settings.

Research is also needed on how best to counsel mothers of infants whose birth test is negative about the need to return for an additional test. Systems to ensure follow-up for a subsequent test require development. This approach may be challenging, especially in the absence of unique patient identifiers across the health service, as subsequent tests in infants born at a single delivery facility would probably occur in many different immunisation clinics.

#### How do we ensure that patients return for results and initiate antiretroviral therapy if indicated?

Irrespective of the timing of testing, there is a need to develop and evaluate systems for ensuring that positive infant results are received and ART is successfully initiated. Interestingly, in the modelling study, changing the timing of a second HIV-PCR test between 6 and 14 weeks of age had only a small effect on the proportion of perinatal infections diagnosed,^30^ whereas the greatest reduction in missed diagnoses (11%) was seen if 100% of caregivers received test results, compared with the assumption of 66% based on previous studies.^35,36^ Data from the Western Cape reassuringly suggest that the proportion of infected infants linked to HIV care has increased from 54% to 71% between 2005 and 2010.^37^ It is likely that there have been further improvements, with rapid alerts of positive tests from laboratories to sub-district PMTCT co-ordinators and immunisation clinics, with follow-up tracing of infants. (Van Niekerk, personal comm.). In contrast, in rural KwaZulu-Natal in 2012, 45% of infants with positive HIV-PCR diagnoses never started ART and a number of challenges in tracing infected infants were identified, highlighting the need for better linkage systems in a range of settings.^38^

Point-of-care (POC) tests could maximise the proportion of infants receiving results, with a number of platforms currently under investigation.^29^ A recent Mozambique study demonstrated 98.5% sensitivity and 99.9% specificity, comparing a POC nucleic acid test implemented in primary care clinics with laboratory tests.^39^ Similar encouraging results were reported for a POC nucleic acid test in Cape Town, South Africa, with overall sensitivity of 97% and specificity of 100% for correctly identifying HIV-infected infants.^40^ Sensitivity was slightly lower (93%) and the test error rate higher (10%) among 90 infants tested at < 7 days old.^40^ Further studies of birth POC tests are ongoing. In addition, the throughput time for a single POC test may limit its use in busier facilities, especially if testing is done on all infants, and not only those at high risk.

Despite the research gaps and challenges described, implementation of routine birth testing in all infants with nearly 100% coverage could itself provide much-needed evidence of the real effectiveness of the PMTCT programme and monitor progress towards virtual elimination of vertical transmission of HIV. Mother to child transmission is probably currently under-estimated both by routine statistics and dedicated studies, as a high proportion of HIV-infected infants may be LTFU or deceased by 6 weeks of age.^2,28^ Similarly, owing to current under-diagnosis of all neonatal infections, the true neonatal mortality in HIV-infected infants is unknown.^15^

### What is the best management for HIV-infected neonates?

#### How soon after birth should antiretroviral therapy be started?

There is a spectrum of arguments favouring early infant ART ranging from the more conventional aims of reducing morbidity and mortality through effective therapy to the potential for modifying persistent HIV to facilitate later treatment-sparing or even eradication strategies.^41,42,43,44,45^ Evidence for the traditional goal of reducing morbidity and mortality includes the high early mortality and rapid disease progression in HIV-infected infants,^46,47,48^ the lack of prognostic markers for mortality in infants^49^ and the substantial reductions in morbidity, mortality, neurodevelopmental delay and other HIV-related complications demonstrated in the CHER randomised controlled trial.^33,50,51^ In addition, cohort studies report better growth, neurocognitive outcomes, immunological response and virological control in infants starting therapy at earlier ages than amongst older infants and children.^45,52,53,54,55,56,57,58,59^

Whilst early virological control in infancy is important for the conventional treatment goals of optimal long-term outcomes on ART, it may also moderate chronic HIV infection by reducing the latent HIV reservoir, paving the way for treatment-sparing strategies.^41,45,53,60^ The case of the ‘Mississippi child’, who received triple therapy within hours of birth with early virological control and subsequent prolonged virological remission off ART,^61^ has sparked interest in treatment-sparing or cure approaches. The final results of the CHER trial found that early therapy followed by interruption after either 40 or 96 weeks on ART had superior clinical and immunological outcomes and less overall time on ART than deferred continuous therapy.^44^ However, it is not known whether longer duration or uninterrupted early therapy would have even better outcomes. In addition, detailed studies on the virological and immunological consequences of interruption, and predictors of the need to restart therapy in the interrupted groups, are still under way. Luzuriaga et al.^53^ recently showed that early treatment at < 2.6 months of age with sustained virological control through to adolescence is associated with limited circulating pro-viral and replication competent virus with continuous decay of viral reservoirs, not seen in children starting ART at older ages. Nevertheless, whilst early virological control in infants may allow for later treatment interruptions or eradication strategies, it does not guarantee prolonged absence of viral rebound, which has occurred within days to weeks of stopping treatment.^62,63^

It is easy to merge the spectrum of arguments in favour of early infant ART into the general dictum ‘the sooner, the better’, especially with a shift towards birth EID testing. Indeed, the rapid disease progression and mortality in HIV-infected infants by 2–3 months of age and high mortality even in the early treatment arm of the CHER trial suggests that ART initiation before a median of 7.4 weeks of age should be beneficial.^33,46,64^ However, we really do not know how soon is soon enough. There is no clear evidence on the optimal timing of ART between birth and 7.4 weeks for either reducing morbidity and mortality on ART, or later treatment-sparing approaches. Importantly, this evidence is also needed for pre-term and low-birthweight infants who comprise a substantial proportion of infants at risk of vertical transmission.^21,65,66^ Of note, low-birthweight infants (< 2kg) were excluded from the CHER study.^33^ In addition, as studies demonstrating benefits of early infant ART to date have not distinguished between in utero and intrapartum infection, the optimal timing of ART initiation in these groups may differ.^47,67^

#### Which regimen should be used in the neonatal period?

The optimal timing of neonatal ART initiation must balance the benefits and risks of early therapy, and hence the lack of appropriate formulations or pharmacokinetic, dosing, safety and effectiveness data for drugs in neonates, especially premature neonates, as outlined in the companion article by Nuttall,^16^ are research gaps. In particular, the use of a nevirapine-based regimen in neonates < 2 weeks of age is a concern with high prevalence of Non-nucleoside reverse-transcriptase inhibitors (NNRTI) resistance, even in the absence of reported PMTCT exposure.^68^ Prevalence of resistance may be even higher than previously reported if more sensitive testing methods are used.^69^ There is mixed evidence on the benefit of more aggressive regimens that hasten virological control, for example four-drug regimens.^54,70,71^ Studies are warranted on the safety and effectiveness of different regimens, including triple-class four-drug regimens and integrase inhibitors. The role of these regimens may be particularly important if the goal of therapy is to allow for later treatment-sparing strategies.^41^ In addition, as adult ART programmes mature, choice of regimen in infants born to mothers failing first-line ART is an emerging research need.

Evidence for which drugs to initiate should consider the likely characteristics and comorbidities in infants infected despite a comprehensive high-coverage PMTCT programme. In a case series of 20 infants initiating ART within the first 6 weeks of life at Rahima Moosa Mother and Child Hospital in Johannesburg, 70% had congenital infections and other illnesses requiring treatment.^72^ Prematurity (70%), low birthweight (50%), and pre-treatment thrombocytopaenia (30%), anaemia (40%) and renal dysfunction (10%) were not uncommon.^72^ Illnesses requiring treatment included congenital pneumonia, congenital syphilis, CMV and TB.^72^ Similar results have been reported from a case series of infants diagnosed at birth at Mowbray Maternity Hospital, Cape Town.^73^ Drug interactions may occur and drugs may require administration via nasogastric or orogastric tubes in sick pre-term infants, affecting dose delivery.^65^

Given the lack of data on many drugs in the neonatal period and probable comorbidities in infected infants, appropriate safety and dose monitoring requires determination. For example, published studies of treated premature neonates have closely monitored drug levels to determine dosing, and the extent to which this is required routinely is unclear.^65^ Similarly, the optimal intensity of safety monitoring is unknown both for sick neonates in hospital as well as for those who are clinically well who could be treated in primary care settings.^74^

#### How do we initiate and retain children on antiretroviral therapy from the neonatal period onwards?

There are substantial challenges with initiating and retaining HIV-infected newborns on treatment. The mothers of HIV-infected infants will frequently have either never accessed PMTCT or been lost to care;^75,76,77^ consequently, the likelihood of poor infant retention and adherence after ART initiation is high. Studies from both wealthy countries^78^ and resource-limited settings^79^ report substantial loss to follow-up and poor adherence on ART in infected infants, despite high coverage of an effective PMTCT programme. In a Johannesburg research cohort of 30 infants initiating ART at a median 16 weeks of age, < 50% were in care 68 weeks later, with four deaths.^79^ The challenges that the caregivers of these infants may already face with accessing and remaining in care may be exacerbated by the tremendous stress experienced with EID.^80,81,82,83^ Significant research gaps include understanding the tensions between maternal needs in the post-partum period, including the psychosocial readiness of the mother to initiate ART for her infant, and how neonatal ART services are delivered. Research is needed to investigate approaches to EID and neonatal ART initiation, such as the use of heath navigators, that best support engagement and long-term retention in care.

## Future directions

As practice changes towards birth diagnosis and early infant ART, there is an opportunity to collect both programme-level and individual patient data to address some of the research questions related to early neonatal HIV care.^84^ Just as studies of the first paediatric ART programmes in resource-limited settings were used to inform treatment guidelines and programme development,^85,86,87^ careful collection and analysis of observational data from PMTCT, EID and neonatal ART programmes will provide valuable evidence to inform neonatal HIV care. Collaborative research across different sites and settings is needed owing to the small numbers of patients at individual sites and transfer of infants from PMTCT programmes to neonatal/paediatric and primary care ART programmes. Such observational research conducted within South Africa and in other resource-limited settings will be relevant both locally and globally.^84^
